# Bis[(*E*)-1-methyl-4-styrylpyridinium] 4-chloro­benzene­sulfonate iodide

**DOI:** 10.1107/S1600536809038896

**Published:** 2009-10-03

**Authors:** Hoong-Kun Fun, Chanasuk Surasit, Kullapa Chanawanno, Suchada Chantrapromma

**Affiliations:** a X-ray Crystallography Unit, School of Physics, Universiti Sains Malaysia, 11800 USM, Penang, Malaysia; b Crystal Materials Research Unit, Department of Chemistry, Faculty of Science, Prince of Songkla University, Hat-Yai, Songkhla 90112, Thailand

## Abstract

In the title compound, 2C_14_H_14_N^+^·C_6_H_4_ClO_3_S^−^·I^−^, each cation exists in an *E* configuration with respect to the ethenyl bond. The dihedral angle between the pyridinium and benzene rings is 3.98 (6)° in one of the cations and 9.88 (7)° in the other. The two cations are arranged in an anti­parallel manner with π–π inter­actions between pyridinium and benzene rings [centroid–centroid distance = 3.5805 (8) Å]. The benzene ring of the anion makes dihedral angles of 61.20 (6) and 64.25 (6)° with the pyridinium rings of the two cations. In the crystal, the cations are stacked in an anti­parallel manner along the *a* axis, while the anions are linked into chains along the same direction. The ions are linked into a three-dimensional network by C—H⋯I and C—H⋯O hydrogen bonds and C—H⋯π inter­actions. The crystal under investigation was an inversion twin, with a ratio of 61.7 (5):38.3 (5) for the two components.

## Related literature

For bond-length data, see: Allen *et al.* (1987 ▶). For general background to non-linear optical materials, see: Lin *et al.* (2002 ▶); Prasad *et al.* (1991 ▶). For related structures, see: Chanawanno *et al.* (2008 ▶); Chantrapromma *et al.* (2007 ▶; 2009 ▶); Fun *et al.* (2009*a* ▶,*b* ▶). For the stability of the temperature controller used in the data collection, see: Cosier & Glazer, (1986 ▶).
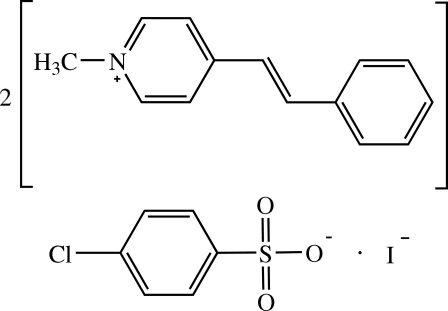


## Experimental

### 

#### Crystal data

2C_14_H_14_N^+^·C_6_H_4_ClO_3_S^−^·I^−^
*M* *_r_* = 711.04
Monoclinic, 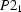

*a* = 8.1103 (1) Å
*b* = 20.5054 (3) Å
*c* = 9.5549 (2) Å
β = 101.799 (1)°
*V* = 1555.45 (4) Å^3^
*Z* = 2
Mo *K*α radiation
μ = 1.22 mm^−1^
*T* = 100 K
0.52 × 0.23 × 0.22 mm

#### Data collection

Bruker APEXII CCD area-detector diffractometer
Absorption correction: multi-scan (*SADABS*; Bruker, 2005 ▶) *T*_min_ = 0.569, *T*_max_ = 0.771
30676 measured reflections
13195 independent reflections
12932 reflections with *I* > 2σ(*I*)
*R*_int_ = 0.020

#### Refinement

*R*[*F* ^2^ > 2σ(*F* ^2^)] = 0.020
*wR*(*F* ^2^) = 0.049
*S* = 1.05
13195 reflections
382 parameters
1 restraint
H-atom parameters constrained
Δρ_max_ = 1.12 e Å^−3^
Δρ_min_ = −0.44 e Å^−3^
Absolute structure: Flack (1983 ▶), 6221 Friedel pairs
Flack parameter: 0.383 (5)

### 

Data collection: *APEX2* (Bruker, 2005 ▶); cell refinement: *SAINT* (Bruker, 2005 ▶); data reduction: *SAINT*; program(s) used to solve structure: *SHELXTL* (Sheldrick, 2008 ▶); program(s) used to refine structure: *SHELXTL*; molecular graphics: *SHELXTL*; software used to prepare material for publication: *SHELXTL* and *PLATON* (Spek, 2009 ▶).

## Supplementary Material

Crystal structure: contains datablocks global, I. DOI: 10.1107/S1600536809038896/ci2919sup1.cif

Structure factors: contains datablocks I. DOI: 10.1107/S1600536809038896/ci2919Isup2.hkl

Additional supplementary materials:  crystallographic information; 3D view; checkCIF report

## Figures and Tables

**Table 1:** Hydrogen-bond geometry (Å, °)

*D*—H⋯*A*	*D*—H	H⋯*A*	*D*⋯*A*	*D*—H⋯*A*
C2—H2*A*⋯O2^i^	0.93	2.36	3.2818 (17)	171
C2*B*—H2*BA*⋯O1^ii^	0.93	2.39	3.2829 (17)	161
C10*B*—H10*B*⋯O2^iii^	0.93	2.54	3.4711 (16)	178
C11*A*—H11*A*⋯O2^ii^	0.93	2.55	3.3109 (16)	139
C7*B*—H7*BA*⋯O2^iii^	0.93	2.53	3.4514 (16)	171
C13*A*—H13*A*⋯O3^iv^	0.93	2.42	2.9706 (16)	118
C14*A*—H14*A*⋯I1^v^	0.96	2.93	3.8718 (13)	168
C14*A*—H14*C*⋯O1^ii^	0.96	2.52	3.1591 (16)	124
C14*A*—H14*B*⋯*Cg*2^i^	0.96	2.71	3.5804 (15)	152
C14*B*—H14*E*⋯*Cg*1^vi^	0.96	2.82	3.5551 (16)	134

Symmetry codes: (i) 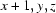; (ii) 
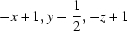
; (iii) 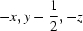; (iv) 
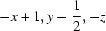
; (v) 
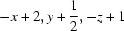
; (vi) 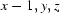. *Cg*1 and *Cg*2 are centroids of the C1*A-*-C6*A* and C1*B-*-C6*B* rings, respectively.

**Fig. 1.**
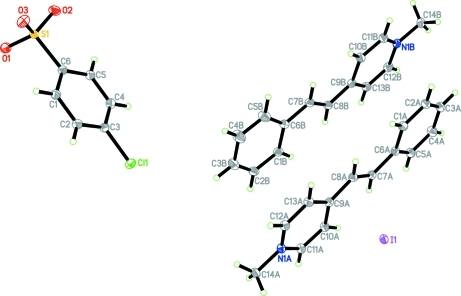
The molecular structure of the title compound, with 50% probability displacement ellipsoids and the atom-numbering scheme.

**Fig. 2.**
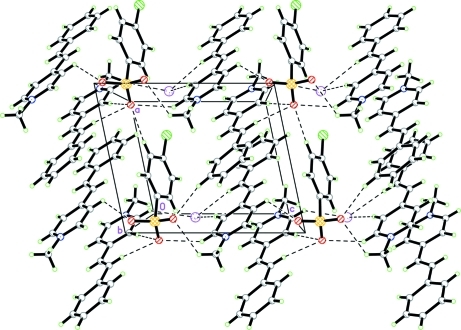
The crystal packing of the title compound viewed down the b axis. C—H···O and C—H···I interactions are shown as dashed lines.

## supplementary crystallographic information

### Comment

There is a considerable interest in the synthesis of new materials with large second-order optical nonlinearities. Such materials require molecular first hyperpolarizability and orientation in a noncentrosymmetric arrangement (Lin *et al.*, 2002; Prasad *et al.* 1991). During the course of our systematic studies of organic NLO materials, we have previously synthesized and reported crystal structures of a number of pyridinium derivatives (Chanawanno *et al.*, 2008; Chantrapromma *et al.*, 2007, 2009; Fun *et al.*, 2009*a*,*b*). Herein we report the crystal structure of the title pyridinium derivative which crystallizes in noncentrosymmetric *P*2_1_ space group and exhibits second-order nonlinear optical properties.

The title molecule consists of two C_14_H_14_N^+^ (*A* and *B*) cations, one C_6_H_4_ClO_3_S^-^ anion and one I^-^ ion (Fig. 1); the two cations exist in an *E* configuration with respect to the C7═C8 ethenyl bond, with a C6–C7–C8–C9 torsion angle of 179.94 (12)° in *A* and 178.99 (12)° in *B*. One cation [*A*] is almost planar while the other [*B*] is slightly twisted; the dihedral angle between the pyridinium and benzene rings is 3.98 (6)° in the cation *A* and 9.88 (7)° in *B*. The orientation of the anion with respect to cations *A* and *B* is indicated by dihedral angles between the benzene ring of the anion and the pyridinium rings of the cations A and B of 61.20 (6) and 64.25 (6)°, respectively. Bond distances in both cations and anion have normal values (Allen *et al.*, 1987) and are comparable with those observed in related structures (Fun *et al.*, 2009*a*,*b*).

In the crystal packing (Fig. 2), all O atoms of the sulfonate group are involved in weak C—H···O interactions (Table 1). The cations are stacked in an antiparallel manner along the *a* axis while the anions are linked into chains along the same direction. The cations are linked to the interstitial I^-^ ions by C—H···I weak interactions and are linked to anionic chains through C—H···O weak interactions (Table 1) forming a three-dimensional network. The crystal structure is further stabilized by C—H···π (Table 1) interactions, and π–π interactions with a Cg1···Cg3 distance of 3.5805 (8) Å (Cg1 and Cg3 are centroids of the C1A–C6A and C9B–C13B/N1B rings respectively. In addition, the crystal structure also shows short C···O [2.9706 (16) Å] and Cl···I [3.5917 (3) Å] contacts.

### Experimental

(*E*)-1-Methyl-4-styrylpyridinium iodide (compound A) was prepared by mixing 1:1:1 molar ratio solutions of 1,4-dimethylpyridinium iodide (2 g, 8.5 mmol), benzaldehyde (0.86 ml, 8.5 mmol) and piperidine (0.84 ml, 8.5 mmol) in methanol (40 ml). The resulting solution was refluxed for 3 h under a nitrogen atmosphere. The yellow solid which formed was filtered and washed with diethyl ether. The title compound was prepared by mixing the compound A (2.75 g, 8.5 mmol) and silver(I) 4-chlorobenzenesulfonate (2.54 g, 8.5 mmol) (Chantrapromma *et al.*, 2007) in methanol (100 ml). The mixture solution was stirred for 30 min, the precipitate of silver iodide which formed was filtered and the filtrate was evaporated to give the title compound as a yellow solid. Yellow needle-shaped single crystals of the title compound suitable for *X*-ray structure determination were recrystallized from a methanol solution by slow evaporation at room temperature over a few weeks (m.p. 468-469 K).

### Refinement

All H atoms were positioned geometrically and allowed to ride on their parent atoms, with d(C-H) = 0.93 Å for aromatic and CH and 0.96 Å for CH_3_ atoms. The *U*_iso_ values were constrained to be 1.5*U*_eq_ of the carrier atom for methyl H atoms and 1.2*U*_eq_ for the remaining H atoms. A rotating group model was used for the methyl groups. The highest residual electron density peak is located at 0.69 Å from I1 and the deepest hole is located at 1.14 Å from I1. The crystal under investigation was an inversion twin, with a ratio of 61.7 (5):38.3 (5) for the two components.

### Crystal data

**Table:**

2C_14_H_14_N^+^·C_6_H_4_ClO_3_S^−^·I^−^	*F*(000) = 720
*M**_r_* = 711.04	*D*_x_ = 1.518 Mg m^−^^3^
Monoclinic, *P*2_1_	Melting point = 468–469 K
Hall symbol: P 2yb	Mo *K*α radiation, λ = 0.71073 Å
*a* = 8.1103 (1) Å	Cell parameters from 13195 reflections
*b* = 20.5054 (3) Å	θ = 2.0–35.0°
*c* = 9.5549 (2) Å	µ = 1.22 mm^−^^1^
β = 101.799 (1)°	*T* = 100 K
*V* = 1555.45 (4) Å^3^	Needle, yellow
*Z* = 2	0.52 × 0.23 × 0.22 mm

### Data collection

**Table:**

Bruker APEXII CCD area-detector diffractometer	13195 independent reflections
Radiation source: sealed tube	12932 reflections with *I* > 2σ(*I*)
graphite	*R*_int_ = 0.020
φ and ω scans	θ_max_ = 35.0°, θ_min_ = 2.0°
Absorption correction: multi-scan (*SADABS*; Bruker, 2005)	*h* = −11→13
*T*_min_ = 0.569, *T*_max_ = 0.771	*k* = −32→33
30676 measured reflections	*l* = −15→15

### Refinement

**Table:**

Refinement on *F*^2^	Secondary atom site location: difference Fourier map
Least-squares matrix: full	Hydrogen site location: inferred from neighbouring sites
*R*[*F*^2^ > 2σ(*F*^2^)] = 0.020	H-atom parameters constrained
*wR*(*F*^2^) = 0.049	*w* = 1/[σ^2^(*F*_o_^2^) + (0.0253*P*)^2^ + 0.205*P*] where *P* = (*F*_o_^2^ + 2*F*_c_^2^)/3
*S* = 1.05	(Δ/σ)_max_ = 0.002
13195 reflections	Δρ_max_ = 1.12 e Å^−^^3^
382 parameters	Δρ_min_ = −0.44 e Å^−^^3^
1 restraint	Absolute structure: Flack (1983), 6221 Friedel pairs
Primary atom site location: structure-invariant direct methods	Flack parameter: 0.383 (5)

### Special details

**Table:**

Experimental. The crystal was placed in the cold stream of an Oxford Cryosystems Cobra open-flow nitrogen cryostat (Cosier & Glazer, 1986) operating at 100.0 (1) K.
Geometry. All esds (except the esd in the dihedral angle between two l.s. planes) are estimated using the full covariance matrix. The cell esds are taken into account individually in the estimation of esds in distances, angles and torsion angles; correlations between esds in cell parameters are only used when they are defined by crystal symmetry. An approximate (isotropic) treatment of cell esds is used for estimating esds involving l.s. planes.
Refinement. Refinement of F^2^ against ALL reflections. The weighted R-factor wR and goodness of fit S are based on F^2^, conventional R-factors R are based on F, with F set to zero for negative F^2^. The threshold expression of F^2^ > 2sigma(F^2^) is used only for calculating R-factors(gt) etc. and is not relevant to the choice of reflections for refinement. R-factors based on F^2^ are statistically about twice as large as those based on F, and R- factors based on ALL data will be even larger.

### Fractional atomic coordinates and isotropic or equivalent isotropic displacement parameters (Å^2^)

**Table:**

	*x*	*y*	*z*	*U*_iso_*/*U*_eq_	
I1	0.971569 (9)	−0.060603 (4)	0.651289 (7)	0.01701 (2)	
Cl1	0.67341 (4)	0.547963 (16)	0.28511 (4)	0.02196 (6)	
S1	0.03835 (4)	0.730613 (14)	0.12930 (3)	0.01312 (5)	
O1	0.06242 (12)	0.77622 (5)	0.24883 (10)	0.01933 (17)	
O2	−0.10740 (12)	0.68847 (5)	0.12227 (11)	0.02079 (18)	
O3	0.04572 (13)	0.76183 (5)	−0.00555 (10)	0.02012 (18)	
C1	0.37016 (15)	0.69739 (6)	0.13653 (14)	0.0164 (2)	
H1A	0.3781	0.7374	0.0925	0.020*	
C2	0.51192 (16)	0.65747 (6)	0.17238 (14)	0.0177 (2)	
H2A	0.6146	0.6704	0.1524	0.021*	
C3	0.49665 (16)	0.59796 (6)	0.23854 (13)	0.0166 (2)	
C4	0.34485 (16)	0.57690 (6)	0.26735 (14)	0.0178 (2)	
H4A	0.3371	0.5367	0.3104	0.021*	
C5	0.20354 (15)	0.61712 (6)	0.23060 (13)	0.0162 (2)	
H5A	0.1006	0.6036	0.2489	0.019*	
C6	0.21641 (14)	0.67745 (6)	0.16659 (12)	0.01420 (19)	
N1A	1.07500 (13)	0.22845 (5)	0.46320 (11)	0.01458 (17)	
C1A	0.48773 (16)	−0.01117 (6)	0.08410 (13)	0.0167 (2)	
H1AA	0.5010	0.0266	0.0337	0.020*	
C2A	0.37605 (13)	−0.05929 (10)	0.02004 (11)	0.01769 (17)	
H2AA	0.3156	−0.0535	−0.0729	0.021*	
C3A	0.35455 (17)	−0.11606 (7)	0.09470 (15)	0.0190 (2)	
H3AA	0.2795	−0.1480	0.0520	0.023*	
C4A	0.44606 (18)	−0.12468 (7)	0.23355 (15)	0.0204 (2)	
H4AA	0.4319	−0.1625	0.2836	0.024*	
C5A	0.55848 (17)	−0.07700 (6)	0.29767 (14)	0.0182 (2)	
H5AA	0.6197	−0.0834	0.3901	0.022*	
C6A	0.58075 (15)	−0.01937 (6)	0.22463 (13)	0.01479 (19)	
C7A	0.70002 (15)	0.02915 (6)	0.29811 (13)	0.01519 (19)	
H7AA	0.7571	0.0188	0.3901	0.018*	
C8A	0.73513 (15)	0.08732 (6)	0.24553 (13)	0.01525 (19)	
H8AA	0.6790	0.0983	0.1536	0.018*	
C9A	0.85493 (14)	0.13424 (6)	0.32263 (12)	0.01350 (18)	
C10A	0.95355 (16)	0.12280 (6)	0.45995 (12)	0.01550 (19)	
H10A	0.9469	0.0829	0.5049	0.019*	
C11A	1.05955 (16)	0.17068 (6)	0.52732 (13)	0.0160 (2)	
H11A	1.1219	0.1631	0.6189	0.019*	
C12A	0.98620 (16)	0.24042 (6)	0.32967 (13)	0.0160 (2)	
H12A	0.9998	0.2798	0.2853	0.019*	
C13A	0.87579 (15)	0.19443 (6)	0.25929 (13)	0.01551 (19)	
H13A	0.8141	0.2035	0.1682	0.019*	
C14A	1.19046 (16)	0.27831 (6)	0.53982 (14)	0.0181 (2)	
H14A	1.1509	0.3209	0.5072	0.027*	
H14B	1.3013	0.2716	0.5215	0.027*	
H14C	1.1943	0.2748	0.6406	0.027*	
N1B	−0.04690 (13)	−0.05269 (6)	0.10397 (12)	0.0163 (2)	
C1B	0.52680 (16)	0.20510 (7)	0.42045 (13)	0.0170 (2)	
H1BA	0.5333	0.1669	0.4738	0.020*	
C2B	0.62871 (17)	0.25784 (7)	0.47254 (14)	0.0197 (2)	
H2BA	0.7030	0.2547	0.5604	0.024*	
C3B	0.61999 (19)	0.31539 (7)	0.39368 (16)	0.0242 (3)	
H3BA	0.6879	0.3507	0.4289	0.029*	
C4B	0.5092 (2)	0.31970 (8)	0.26224 (18)	0.0299 (3)	
H4BA	0.5033	0.3580	0.2093	0.036*	
C5B	0.4076 (2)	0.26720 (7)	0.20969 (16)	0.0251 (3)	
H5BA	0.3341	0.2706	0.1215	0.030*	
C6B	0.41412 (15)	0.20902 (6)	0.28785 (13)	0.0160 (2)	
C7B	0.30241 (15)	0.15565 (6)	0.22664 (13)	0.0163 (2)	
H7BA	0.2387	0.1620	0.1351	0.020*	
C8B	0.28271 (15)	0.09829 (6)	0.28959 (13)	0.0160 (2)	
H8BA	0.3467	0.0907	0.3806	0.019*	
C9B	0.16733 (15)	0.04727 (6)	0.22408 (13)	0.01487 (19)	
C10B	0.07550 (16)	0.05083 (6)	0.08199 (13)	0.0167 (2)	
H10B	0.0861	0.0872	0.0263	0.020*	
C11B	−0.02934 (16)	0.00070 (6)	0.02581 (13)	0.0173 (2)	
H11B	−0.0895	0.0036	−0.0679	0.021*	
C12B	0.03766 (14)	−0.05758 (10)	0.24086 (12)	0.01881 (19)	
H12B	0.0237	−0.0944	0.2941	0.023*	
C13B	0.14428 (16)	−0.00860 (6)	0.30211 (13)	0.0178 (2)	
H13B	0.2017	−0.0126	0.3965	0.021*	
C14B	−0.15834 (18)	−0.10610 (7)	0.03712 (16)	0.0211 (2)	
H14D	−0.1210	−0.1215	−0.0460	0.032*	
H14E	−0.2717	−0.0902	0.0098	0.032*	
H14F	−0.1546	−0.1412	0.1042	0.032*	

### Atomic displacement parameters (Å^2^)

**Table:**

	*U*^11^	*U*^22^	*U*^33^	*U*^12^	*U*^13^	*U*^23^
I1	0.01966 (3)	0.01429 (3)	0.01623 (3)	−0.00068 (3)	0.00170 (2)	−0.00045 (3)
Cl1	0.01781 (12)	0.01998 (13)	0.02531 (14)	0.00622 (10)	−0.00208 (10)	−0.00453 (11)
S1	0.01285 (11)	0.01510 (12)	0.01106 (10)	0.00126 (9)	0.00161 (8)	−0.00187 (9)
O1	0.0200 (4)	0.0214 (4)	0.0153 (4)	0.0047 (3)	0.0005 (3)	−0.0054 (3)
O2	0.0129 (4)	0.0241 (5)	0.0247 (5)	−0.0027 (3)	0.0023 (3)	−0.0021 (4)
O3	0.0232 (4)	0.0231 (5)	0.0149 (4)	0.0065 (4)	0.0058 (3)	0.0023 (3)
C1	0.0142 (4)	0.0151 (5)	0.0202 (5)	−0.0006 (4)	0.0041 (4)	−0.0008 (4)
C2	0.0135 (4)	0.0182 (5)	0.0211 (5)	0.0002 (4)	0.0028 (4)	−0.0024 (4)
C3	0.0152 (5)	0.0163 (5)	0.0168 (5)	0.0029 (4)	−0.0005 (4)	−0.0044 (4)
C4	0.0185 (5)	0.0150 (5)	0.0189 (5)	0.0005 (4)	0.0012 (4)	−0.0005 (4)
C5	0.0147 (5)	0.0157 (5)	0.0180 (5)	0.0000 (4)	0.0030 (4)	−0.0011 (4)
C6	0.0120 (4)	0.0151 (5)	0.0152 (5)	0.0007 (3)	0.0021 (3)	−0.0024 (4)
N1A	0.0161 (4)	0.0126 (4)	0.0150 (4)	−0.0004 (3)	0.0032 (3)	−0.0001 (3)
C1A	0.0178 (5)	0.0156 (5)	0.0163 (5)	0.0002 (4)	0.0026 (4)	0.0013 (4)
C2A	0.0180 (4)	0.0169 (4)	0.0172 (4)	−0.0002 (6)	0.0014 (3)	−0.0014 (6)
C3A	0.0186 (5)	0.0161 (5)	0.0219 (5)	−0.0018 (4)	0.0028 (4)	−0.0014 (4)
C4A	0.0227 (6)	0.0161 (5)	0.0220 (6)	−0.0035 (4)	0.0035 (4)	0.0028 (4)
C5A	0.0196 (5)	0.0159 (5)	0.0184 (5)	−0.0002 (4)	0.0021 (4)	0.0018 (4)
C6A	0.0139 (4)	0.0141 (5)	0.0162 (5)	−0.0001 (4)	0.0028 (4)	0.0000 (4)
C7A	0.0151 (5)	0.0144 (5)	0.0157 (5)	0.0006 (4)	0.0023 (4)	−0.0002 (4)
C8A	0.0147 (4)	0.0154 (5)	0.0148 (4)	0.0002 (4)	0.0011 (4)	0.0001 (4)
C9A	0.0142 (4)	0.0140 (5)	0.0130 (4)	0.0005 (3)	0.0046 (3)	0.0006 (3)
C10A	0.0186 (5)	0.0137 (5)	0.0139 (4)	−0.0005 (4)	0.0026 (4)	0.0015 (4)
C11A	0.0200 (5)	0.0146 (5)	0.0130 (4)	0.0004 (4)	0.0025 (4)	0.0014 (4)
C12A	0.0179 (5)	0.0148 (5)	0.0153 (5)	−0.0001 (4)	0.0037 (4)	0.0026 (4)
C13A	0.0166 (5)	0.0153 (5)	0.0144 (4)	−0.0004 (4)	0.0025 (4)	0.0030 (4)
C14A	0.0182 (5)	0.0157 (5)	0.0205 (5)	−0.0032 (4)	0.0040 (4)	−0.0026 (4)
N1B	0.0153 (4)	0.0137 (6)	0.0201 (4)	0.0012 (3)	0.0042 (3)	−0.0024 (4)
C1B	0.0158 (5)	0.0200 (5)	0.0145 (5)	0.0018 (4)	0.0016 (4)	−0.0005 (4)
C2B	0.0157 (5)	0.0248 (6)	0.0179 (5)	0.0003 (4)	0.0020 (4)	−0.0047 (4)
C3B	0.0228 (6)	0.0229 (6)	0.0260 (6)	−0.0064 (5)	0.0032 (5)	−0.0038 (5)
C4B	0.0351 (8)	0.0214 (7)	0.0297 (7)	−0.0089 (6)	−0.0018 (6)	0.0058 (5)
C5B	0.0270 (7)	0.0234 (6)	0.0214 (6)	−0.0048 (5)	−0.0032 (5)	0.0053 (5)
C6B	0.0145 (5)	0.0178 (5)	0.0153 (5)	−0.0007 (4)	0.0021 (4)	0.0000 (4)
C7B	0.0152 (4)	0.0181 (5)	0.0146 (4)	0.0003 (4)	0.0009 (4)	−0.0004 (4)
C8B	0.0149 (5)	0.0175 (5)	0.0150 (5)	0.0007 (4)	0.0018 (4)	−0.0004 (4)
C9B	0.0144 (4)	0.0149 (5)	0.0154 (5)	0.0013 (4)	0.0033 (4)	−0.0001 (4)
C10B	0.0184 (5)	0.0161 (5)	0.0153 (5)	−0.0004 (4)	0.0025 (4)	0.0004 (4)
C11B	0.0185 (5)	0.0165 (5)	0.0163 (5)	0.0004 (4)	0.0023 (4)	−0.0008 (4)
C12B	0.0196 (4)	0.0164 (5)	0.0203 (4)	0.0006 (6)	0.0038 (3)	0.0038 (6)
C13B	0.0191 (5)	0.0175 (5)	0.0163 (5)	0.0008 (4)	0.0028 (4)	0.0015 (4)
C14B	0.0206 (5)	0.0154 (5)	0.0267 (6)	−0.0006 (4)	0.0032 (5)	−0.0049 (4)

### Geometric parameters (Å, °)

**Table:**

Cl1—C3	1.7445 (13)	C11A—H11A	0.93
S1—O3	1.4509 (10)	C12A—C13A	1.3775 (18)
S1—O2	1.4547 (10)	C12A—H12A	0.93
S1—O1	1.4581 (10)	C13A—H13A	0.93
S1—C6	1.7861 (12)	C14A—H14A	0.96
C1—C2	1.3958 (18)	C14A—H14B	0.96
C1—C6	1.3968 (17)	C14A—H14C	0.96
C1—H1A	0.93	N1B—C11B	1.3490 (18)
C2—C3	1.3914 (19)	N1B—C12B	1.3507 (15)
C2—H2A	0.93	N1B—C14B	1.4793 (17)
C3—C4	1.3844 (19)	C1B—C2B	1.3901 (19)
C4—C5	1.3972 (18)	C1B—C6B	1.4047 (16)
C4—H4A	0.93	C1B—H1BA	0.93
C5—C6	1.3935 (18)	C2B—C3B	1.394 (2)
C5—H5A	0.93	C2B—H2BA	0.93
N1A—C11A	1.3512 (16)	C3B—C4B	1.389 (2)
N1A—C12A	1.3531 (15)	C3B—H3BA	0.93
N1A—C14A	1.4758 (16)	C4B—C5B	1.386 (2)
C1A—C2A	1.394 (2)	C4B—H4BA	0.93
C1A—C6A	1.4093 (17)	C5B—C6B	1.4026 (19)
C1A—H1AA	0.93	C5B—H5BA	0.93
C2A—C3A	1.395 (2)	C6B—C7B	1.4643 (18)
C2A—H2AA	0.93	C7B—C8B	1.3454 (18)
C3A—C4A	1.3927 (19)	C7B—H7BA	0.93
C3A—H3AA	0.93	C8B—C9B	1.4568 (17)
C4A—C5A	1.3907 (18)	C8B—H8BA	0.93
C4A—H4AA	0.93	C9B—C13B	1.4002 (18)
C5A—C6A	1.4027 (18)	C9B—C10B	1.4105 (17)
C5A—H5AA	0.93	C10B—C11B	1.3716 (18)
C6A—C7A	1.4632 (17)	C10B—H10B	0.93
C7A—C8A	1.3470 (17)	C11B—H11B	0.93
C7A—H7AA	0.93	C12B—C13B	1.375 (2)
C8A—C9A	1.4559 (16)	C12B—H12B	0.93
C8A—H8AA	0.93	C13B—H13B	0.93
C9A—C13A	1.4000 (17)	C14B—H14D	0.96
C9A—C10A	1.4096 (16)	C14B—H14E	0.96
C10A—C11A	1.3751 (17)	C14B—H14F	0.96
C10A—H10A	0.93		
			
O3—S1—O2	113.69 (6)	N1A—C12A—C13A	120.24 (11)
O3—S1—O1	112.92 (6)	N1A—C12A—H12A	119.9
O2—S1—O1	113.24 (6)	C13A—C12A—H12A	119.9
O3—S1—C6	105.54 (6)	C12A—C13A—C9A	121.10 (11)
O2—S1—C6	105.24 (6)	C12A—C13A—H13A	119.4
O1—S1—C6	105.18 (5)	C9A—C13A—H13A	119.4
C2—C1—C6	120.20 (12)	N1A—C14A—H14A	109.5
C2—C1—H1A	119.9	N1A—C14A—H14B	109.5
C6—C1—H1A	119.9	H14A—C14A—H14B	109.5
C3—C2—C1	118.72 (12)	N1A—C14A—H14C	109.5
C3—C2—H2A	120.6	H14A—C14A—H14C	109.5
C1—C2—H2A	120.6	H14B—C14A—H14C	109.5
C4—C3—C2	121.96 (12)	C11B—N1B—C12B	120.41 (12)
C4—C3—Cl1	118.93 (10)	C11B—N1B—C14B	119.09 (11)
C2—C3—Cl1	119.10 (10)	C12B—N1B—C14B	120.50 (12)
C3—C4—C5	118.88 (12)	C2B—C1B—C6B	120.52 (12)
C3—C4—H4A	120.6	C2B—C1B—H1BA	119.7
C5—C4—H4A	120.6	C6B—C1B—H1BA	119.7
C6—C5—C4	120.21 (12)	C1B—C2B—C3B	120.29 (12)
C6—C5—H5A	119.9	C1B—C2B—H2BA	119.9
C4—C5—H5A	119.9	C3B—C2B—H2BA	119.9
C5—C6—C1	120.01 (11)	C4B—C3B—C2B	119.62 (13)
C5—C6—S1	119.96 (9)	C4B—C3B—H3BA	120.2
C1—C6—S1	119.98 (9)	C2B—C3B—H3BA	120.2
C11A—N1A—C12A	120.38 (11)	C5B—C4B—C3B	120.32 (14)
C11A—N1A—C14A	119.39 (10)	C5B—C4B—H4BA	119.8
C12A—N1A—C14A	120.23 (10)	C3B—C4B—H4BA	119.8
C2A—C1A—C6A	120.48 (12)	C4B—C5B—C6B	120.84 (13)
C2A—C1A—H1AA	119.8	C4B—C5B—H5BA	119.6
C6A—C1A—H1AA	119.8	C6B—C5B—H5BA	119.6
C1A—C2A—C3A	120.29 (10)	C5B—C6B—C1B	118.41 (12)
C1A—C2A—H2AA	119.9	C5B—C6B—C7B	118.04 (11)
C3A—C2A—H2AA	119.9	C1B—C6B—C7B	123.55 (11)
C4A—C3A—C2A	119.68 (12)	C8B—C7B—C6B	126.53 (11)
C4A—C3A—H3AA	120.2	C8B—C7B—H7BA	116.7
C2A—C3A—H3AA	120.2	C6B—C7B—H7BA	116.7
C5A—C4A—C3A	120.27 (12)	C7B—C8B—C9B	124.07 (11)
C5A—C4A—H4AA	119.9	C7B—C8B—H8BA	118.0
C3A—C4A—H4AA	119.9	C9B—C8B—H8BA	118.0
C4A—C5A—C6A	120.84 (12)	C13B—C9B—C10B	116.95 (11)
C4A—C5A—H5AA	119.6	C13B—C9B—C8B	120.18 (11)
C6A—C5A—H5AA	119.6	C10B—C9B—C8B	122.87 (11)
C5A—C6A—C1A	118.44 (11)	C11B—C10B—C9B	120.06 (12)
C5A—C6A—C7A	118.29 (11)	C11B—C10B—H10B	120.0
C1A—C6A—C7A	123.27 (11)	C9B—C10B—H10B	120.0
C8A—C7A—C6A	126.34 (11)	N1B—C11B—C10B	121.25 (11)
C8A—C7A—H7AA	116.8	N1B—C11B—H11B	119.4
C6A—C7A—H7AA	116.8	C10B—C11B—H11B	119.4
C7A—C8A—C9A	124.51 (11)	N1B—C12B—C13B	120.56 (14)
C7A—C8A—H8AA	117.7	N1B—C12B—H12B	119.7
C9A—C8A—H8AA	117.7	C13B—C12B—H12B	119.7
C13A—C9A—C10A	116.96 (11)	C12B—C13B—C9B	120.77 (12)
C13A—C9A—C8A	119.41 (10)	C12B—C13B—H13B	119.6
C10A—C9A—C8A	123.64 (11)	C9B—C13B—H13B	119.6
C11A—C10A—C9A	119.89 (11)	N1B—C14B—H14D	109.5
C11A—C10A—H10A	120.1	N1B—C14B—H14E	109.5
C9A—C10A—H10A	120.1	H14D—C14B—H14E	109.5
N1A—C11A—C10A	121.38 (11)	N1B—C14B—H14F	109.5
N1A—C11A—H11A	119.3	H14D—C14B—H14F	109.5
C10A—C11A—H11A	119.3	H14E—C14B—H14F	109.5
			
C6—C1—C2—C3	−0.17 (18)	C12A—N1A—C11A—C10A	−0.33 (19)
C1—C2—C3—C4	1.13 (19)	C14A—N1A—C11A—C10A	−179.97 (12)
C1—C2—C3—Cl1	−179.50 (10)	C9A—C10A—C11A—N1A	−1.70 (19)
C2—C3—C4—C5	−0.92 (19)	C11A—N1A—C12A—C13A	1.72 (18)
Cl1—C3—C4—C5	179.71 (9)	C14A—N1A—C12A—C13A	−178.65 (12)
C3—C4—C5—C6	−0.26 (18)	N1A—C12A—C13A—C9A	−1.06 (19)
C4—C5—C6—C1	1.19 (18)	C10A—C9A—C13A—C12A	−0.91 (18)
C4—C5—C6—S1	−176.40 (9)	C8A—C9A—C13A—C12A	179.10 (12)
C2—C1—C6—C5	−0.97 (18)	C6B—C1B—C2B—C3B	0.1 (2)
C2—C1—C6—S1	176.61 (10)	C1B—C2B—C3B—C4B	−0.3 (2)
O3—S1—C6—C5	−143.17 (10)	C2B—C3B—C4B—C5B	0.1 (3)
O2—S1—C6—C5	−22.64 (11)	C3B—C4B—C5B—C6B	0.1 (3)
O1—S1—C6—C5	97.21 (11)	C4B—C5B—C6B—C1B	−0.3 (2)
O3—S1—C6—C1	39.25 (11)	C4B—C5B—C6B—C7B	179.55 (15)
O2—S1—C6—C1	159.77 (10)	C2B—C1B—C6B—C5B	0.2 (2)
O1—S1—C6—C1	−80.38 (11)	C2B—C1B—C6B—C7B	−179.66 (12)
C6A—C1A—C2A—C3A	0.28 (19)	C5B—C6B—C7B—C8B	−175.19 (14)
C1A—C2A—C3A—C4A	−0.4 (2)	C1B—C6B—C7B—C8B	4.6 (2)
C2A—C3A—C4A—C5A	0.0 (2)	C6B—C7B—C8B—C9B	178.99 (12)
C3A—C4A—C5A—C6A	0.5 (2)	C7B—C8B—C9B—C13B	−174.52 (13)
C4A—C5A—C6A—C1A	−0.63 (19)	C7B—C8B—C9B—C10B	5.9 (2)
C4A—C5A—C6A—C7A	179.60 (12)	C13B—C9B—C10B—C11B	−0.56 (18)
C2A—C1A—C6A—C5A	0.22 (19)	C8B—C9B—C10B—C11B	179.04 (12)
C2A—C1A—C6A—C7A	179.98 (12)	C12B—N1B—C11B—C10B	0.77 (19)
C5A—C6A—C7A—C8A	−178.90 (13)	C14B—N1B—C11B—C10B	−178.48 (12)
C1A—C6A—C7A—C8A	1.3 (2)	C9B—C10B—C11B—N1B	−0.10 (19)
C6A—C7A—C8A—C9A	179.94 (12)	C11B—N1B—C12B—C13B	−0.73 (19)
C7A—C8A—C9A—C13A	−177.28 (12)	C14B—N1B—C12B—C13B	178.51 (12)
C7A—C8A—C9A—C10A	2.7 (2)	N1B—C12B—C13B—C9B	0.0 (2)
C13A—C9A—C10A—C11A	2.25 (18)	C10B—C9B—C13B—C12B	0.59 (19)
C8A—C9A—C10A—C11A	−177.76 (12)	C8B—C9B—C13B—C12B	−179.02 (12)

### Hydrogen-bond geometry (Å, °)

**Table:**

*D*—H···*A*	*D*—H	H···*A*	*D*···*A*	*D*—H···*A*
C2—H2A···O2^i^	0.93	2.36	3.2818 (17)	171
C2B—H2BA···O1^ii^	0.93	2.39	3.2829 (17)	161
C10B—H10B···O2^iii^	0.93	2.54	3.4711 (16)	178
C11A—H11A···O2^ii^	0.93	2.55	3.3109 (16)	139
C7B—H7BA···O2^iii^	0.93	2.53	3.4514 (16)	171
C13A—H13A···O3^iv^	0.93	2.42	2.9706 (16)	118
C14A—H14A···I1^v^	0.96	2.93	3.8718 (13)	168
C14A—H14C···O1^ii^	0.96	2.52	3.1591 (16)	124
C14A—H14B···Cg2^i^	0.96	2.71	3.5804 (15)	152
C14B—H14E···Cg1^vi^	0.96	2.82	3.5551 (16)	134

Symmetry codes: (i) *x*+1, *y*, *z*; (ii) −*x*+1, *y*−1/2, −*z*+1; (iii) −*x*, *y*−1/2, −*z*; (iv) −*x*+1, *y*−1/2, −*z*; (v) −*x*+2, *y*+1/2, −*z*+1; (vi) *x*−1, *y*, *z*.
